# Poster Session I - A99 REMIFENTANIL’S EFFECT ON PROPOFOL SEDATION IN MARIJUANA USERS UNDERGOING ESOPHAGODUODENOSCOPY

**DOI:** 10.1093/jcag/gwaf042.099

**Published:** 2026-02-13

**Authors:** S Ostrovski, W M Hopman, V Ginzburg

**Affiliations:** Medicine, University of Toronto, Toronto, ON, Canada; Queen’s University, Kingston, ON, Canada; Medicine, University of Toronto, Toronto, ON, Canada

## Abstract

**Background:**

The legalization of recreational marijuana in Canada has led to a sharp rise in use, with more than one in four adults reporting use in the past year. This growing trend presents new challenges for anesthesia providers, as marijuana use has been associated with altered responses to standard sedative medications. Short procedures performed in ambulatory settings, such as esophagogastroduodenoscopy (EGD), rely on precise sedation practices to ensure patient safety and rapid recovery. However, marijuana users often require higher anesthetic doses to achieve adequate sedation, increasing the risk of complications, prolonging recovery times, and straining clinical efficiency. As this population continues to grow, the development of evidence-based strategies to optimize sedation for marijuana users has become an urgent clinical priority. One promising approach involves supplementing propofol with remifentanil, a short-acting opioid that may enhance sedation effectiveness and reduce anesthetic burden.

**Aims:**

To evaluate whether the concurrent administration of remifentanil with propofol can reduce the total propofol dose required for adequate sedation in marijuana-using patients undergoing EGD.

**Methods:**

A retrospective cohort study was conducted using medical records from Oak Ridges Endoscopy Center (2014–2024). Patients were divided into three groups: (1) baseline: non-users receiving propofol only (n = 97), (2) control: marijuana users receiving propofol only (n = 120), and (3) experimental: marijuana users receiving propofol with remifentanil (n = 115). Patients with hepatic or renal impairment, morbid obesity, or chronic benzodiazepine use were excluded. The mean propofol doses across the three study groups were compared. A multivariable linear regression was conducted to account for potential confounders.

**Results:**

Significant differences in propofol dosing were observed between groups (p < 0.001). Marijuana users required substantially higher doses of propofol (mean 224.3 mg ± 3.9) compared to non-users (192.6 mg ± 3.9). The addition of remifentanil reduced the mean propofol dose to 146.7 mg ± 4.4, effectively offsetting the increased requirements observed in marijuana users. Regression analysis confirmed remifentanil use as an independent predictor of reduced propofol dosing.

**Conclusions:**

As marijuana use becomes more common, anesthesia providers must adapt sedation practices to meet the needs of the changing patient population. This study demonstrates that combining remifentanil with propofol reduces anesthetic requirements among marijuana users, improving procedural safety and efficiency. The remifentanil-propofol regimen represents a practical, evidence-based strategy for optimizing sedation in outpatient endoscopy and could serve as a foundation for updated clinical guidelines.

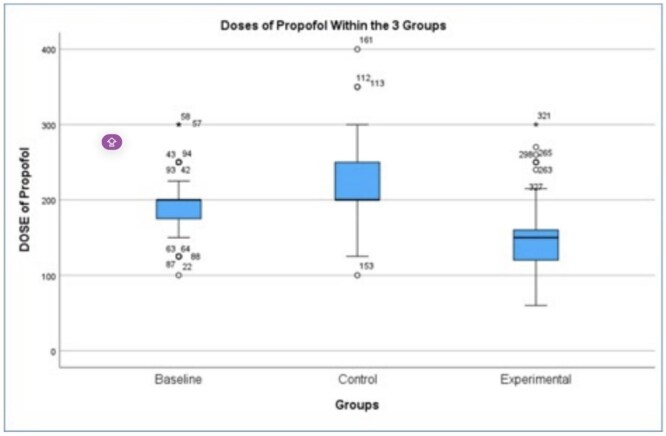

**Funding Agencies:**

None

